# Multiplex NanoSPR Molecular Biosensor for Blood Cytokine Monitoring

**DOI:** 10.34172/apb.2022.046

**Published:** 2021-09-29

**Authors:** Agha Zeeshan Mirza

**Affiliations:** Department of Chemistry, University of Karachi, Karachi, Pakistan

**Keywords:** Cytokines, Multiplex, Diagnosis, NanoSPR

## Abstract

Cytokines, as protein biomarkers, have essential functions in the diagnosis, identification, and healing of a broad range of syndromes. For the specific and accurate monitoring of immune conditions, which change rapidly throughout the duration of disease, sophisticated sensors for detecting cytokines are essential and will assist in clinical testing and studies of various diseases. The present manuscript briefly discusses fundamental principles applied to the development of tools for cytokine detection and new biomarker development. The latest developments in the technologies for highly sensitive and multiplexed cytokine quantification, with current detection capabilities across a broad, vibrant array, are also discussed. Finally, nanomaterial-based cytokine sensors, currently considered new approaches, are presented from the perspective of optimizing the sensitivity and multiplexity of cytokine detection.

## Introduction

Cytokines are part of physiological processes and alter numerous significant characteristics of the inflammatory system. They are small peptides and a necessary component of the host response to injury and stimulation. Cytokines act in autocrine, paracrine, and endocrine manners; hence, the alteration of cytokine profiles in circulation often indicates specific disease conditions. It has been shown that cancer can be promoted by inflammation and infections by creating a tumor-supporting microenvironment that stimulates the neoplastic progression. Cytokines that stimulate innateimmune cells are responsible for tumor growth and progression. Other cytokines, which are produced by inflammatory cells, can limit tumor growth^1^ and can serve as early markers for the detection of cancer.^2^ Cytokine profiling is also useful in predicting serious side effects of cancer treatment, such as severe lung injury from radiation therapy.^2^ In addition, cytokines can be very useful as surrogate markers to assess the response to cancer therapy, especially immunotherapy, and are likely to be used as intermediate markers to help prioritize agents for testing in prospective randomized Phase III trials.

Most cytokines are circulated at extremely low levels under normal conditions ( < 10 pg/mL, i.e., ~0.5-5 pM),^3^ which is much below the detection limits of most assays (cytometry, ELISA, bioassays, GC-MS, and immunoproteomics), or their detection steps are very cumbersome. An ideal cytokine assay for diagnostic purposes should meet the following requirements: (1) the assay should be able to detect cytokines at biologically meaningful levels, i.e., at nanograms per milliliter, in the blood; (2) the assay must be able to rule out the interference of noncytokine agents, such as proteins/peptides, in a biological fluid, i.e., in the serum or blood; (3) the assay must require minimum or no sample preparation steps to detect cytokines and meet the requirements for clinical diagnostics; (4) the assay response time must be rapid, within seconds to minutes; (5) the assay should be translatable to point-of-care use; (6) the assay must be cost-effective; and (7) the assay must be easy to use.

For diagnostic purposes, sometimes, the key requirement is not the absolute quantification but rather a rapid evaluation of a cytokine panel (usually 4-8 cytokines) as markers associated with a specific disease or to diagnose the nature of the condition^4^; hence, multiplex detection will be invaluable. Based on the current developments in the field of nanotechnology, the detection of cytokines takes advantage of various forms of nanomaterials for enhanced sensing capabilities. Owing to their reduced dimensions, nanomaterials have been established to display special and unique optical properties that can be used for qualitative and quantitative analyses of cytokines.^5^ The cytokine markers chosen for multiplex detection are tumour necrosis factor α (TNF-α), interleukin-2 (IL-2), IL-4, IL-6, IL-8, IL-10, IL-12, and IFN-γ. IL-8 has been reported to be a good marker for hepatocellular carcinoma^2^; the elevations of IL-6 and TNF-α have been correlated with chronic fatigue in breast cancer survivors^6^; the elevations of TNF-α, IL-4, IL-6, IL-10, IL-12, and INF-γ have been observed in sepsis, and those of IL-2 and IFN-γ have been observed in both chronic lung inflammation and bowel inflammation.^4^

## Multiplex NanoSPR biosensor

**Figure 1:**
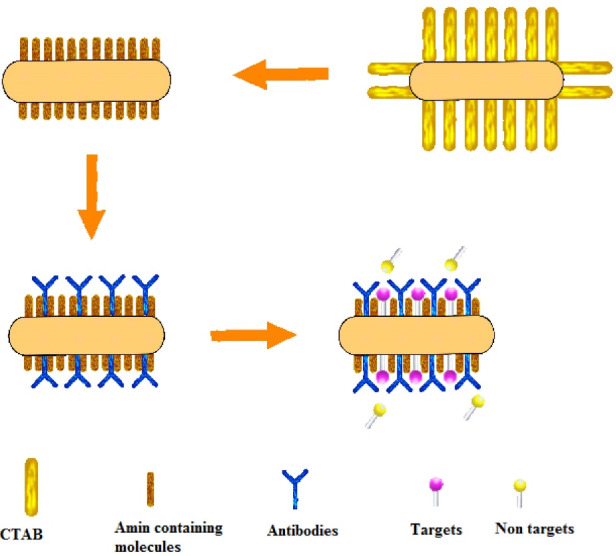
Schematic representation of functionalization procedure.

The plasmonic properties of noble metal films are used for surface plasmon resonance (SPR) -based biosensors, and SPR is notably becoming more relevant for use in biosensor applications. These biosensors are comprehensively investigated owing to the simplicity of detecting visible color changes. Gold nanorods and nanoparticles have numerous distinctive characteristics, which have been investigated for potential relevance to biomolecular detection,^7,8^ and shifts in both transverse and longitudinal surface plasmon resonance were observed in terms of the intensity and wavelength due to chemical functionalization.^9^ This biosensor creates a chemically active group, which is able to attach drug molecules and antibodies to obtain molecular probes.^10,11^ Multiplex sensing has long been established based on distinct responses of the plasmon spectra of these probes to their targets and single-receptor kinetics through the binding with antibodies, viruses, etc. A functionalization procedure was shown to minimize nonspecific binding^12^ (Figure 1).

Simultaneous detection of nine different respiratory viruses, including severe acute respiratory syndrome coronavirus (SARS), was also reported. Biotin was used to increase the sensitivity, while streptavidin induction was responsible for signal amplification.^13^ Similar multiplex immunoassays of a complex serum matrix have been reported to detect six cytokines (up to a concentration of 5–20 pg/mL) on a single device chip, with an optical biosensor device using antibody conjugation of gold nanorods in a microfluidic channel array with 480 nanoplasmonic sensing spots.^14^ This type of assay is significant for immune monitoring in infants and neonates with infectious diseases, as it is complicated to obtain their blood in high quantities.^14^ An ultrasensitive biosensor has been developed using gold nanorings and NIR (near-infrared extinction) localized surface plasmon resonance (LSPR).^15^ Pathogens like thyroglobulin and glycoprotein detections were also reported using a gold nanorod biosensor.^16^ Similarly, to indicates the active viral replication of the hepatitis B virus, a gold nanorods biosensor reported to detected hepatitis B surface antigen (HBsAg) up to 0.01 to 1 IU/mL response range.^17^ In a few reports, the detection limit was estimated to attain femtomolar levels^18^ (Table 1).

**Table 1:** Multiplex biosensors

**Type**	**Targets**	**Detection limits**	**References**
Gold NR	Goat anti-human IgG1 Fab		12
	Rabbit antimouse IgG1 Fab		
	Rabbit anti-sheep IgG (H+L)		
Gold NR	Interleukin-2 (IL-2)	20.56*	14
	Interleukin-4 (IL-4)	4.60	
	Interleukin-6 (IL-6)	11.29	
	Interleukin-10 (IL-10)	10.97	
	Interferon-γ (IFN-γ)	6.46	
	Tumor necrosis factor α (TNF-α)	11.43	
Gold NR	Thyroglobulin		16
	glycoprotein		
Gold NR	Hepatitis B surface antigen (HBsAg)	0.01**	17
Gold NR	Goat anti-human IgG	92.32^	18
	Goat anti-rabbit IgG	19.14	
	goat antimouse IgG	15.86	
Gold NR	*E. coli*	1–10^^	19
	*S. typhimurium*	1–10	
Gold nanoislands	RdRp-COVID	0.22±0.08^+^	20
	ORF1ab-COVID	0.22±0.08	
	E genes from SARS-Cov-2	0.22±0.08	

*pgmL^-1^, **IUmL^-1^, ^nm, ^^cfumL^-1^, +pM

## Clinical COVID-19 diagnosis

The severity of SARS-CoV-2 infection is considered to be due to the intense generation of proinflammatory cytokines, known as a “cytokine storm”, although exact pathophysiology and treatment are still uncertain.^19-21^ SARS-CoV-2 possesses single-stranded, positive-sense RNA and belongs to the family of betacoronaviruses; inherent resistance against SARS-Cov-2 appears essential to manage and control viral infection. Hydroxychloroquine, as well as IL-6 and IL-1 antagonists, may be considered, while IFN-α, lopinavir/ritonavir, ribavirin, and Arbidol^®^ are recommended as antiviral therapies and for the treatment of COVID-19.^20-22^ Currently, a reverse transcription-polymerase chain reaction (RT-PCR) is used as a reference test for the diagnosis of COVID-19. In the initial period of the outbreak of the novel virus, several false-positive or negative cases were reported. For the clinical COVID-19 diagnosis, a dual-functional plasmonic biosensor containing two-dimensional gold nanoislands was functionalized through corresponding DNA receptors with nucleic acid hybridization. A highly sensitive LSPR biosensor showed a lower limit of detection (at a concentration of 0.22 pM).^23^ A field-effect transistor sensor, coated with graphene sheets, has recently been reported to detect the SARS-CoV-2 spike protein at concentrations of 100 fg/mL in the clinical transport medium and 1.6 × 10^1^ pfu/mL in the culture medium.^24^

## Conclusion

As a result of the prompt rise in the rate of human SARS-CoV-2 disease, the World Health Organization confirmed the COVID-19 epidemic as a pandemic. Nevertheless, there are no specific drugs or vaccines available for COVID-19, while early identification and diagnosis are essential to control the outbreak. This paper aimed to briefly describe the current development of a novel, yet simple, multiplex molecular technology, including the efforts to develop a highly sensitive immunological nanoSPR molecular probe concept, based on gold nanorods, for the fast, accurate and sensitive 8-plex cytokine monitoring. The Multiplex nanoSPR molecular biosensor holds a bright future in the early assessment of disease with high sensitivity and accuracy.

## Acknowledgments

The author is thankful to Dr. Hina Shamshad, University of Karachi for his help with the manuscript.

## Ethical Issues

Not applicable.

## Conflict of Interest

No potential conflict of interest relevant to this article was reported.
